# Materials Prepared via Pickering Emulsions Stabilized by Graphene Oxide: Overview and Prospects

**DOI:** 10.3390/ma18204790

**Published:** 2025-10-20

**Authors:** Manman Liu, Wenle Zhu, Huili Wang

**Affiliations:** 1 State Key Laboratory of Green Papermaking and Resource Recycling, Qilu University of Technology, Jinan 250353, China; 2 School of Chemistry and Materials Science, South-Central Minzu University, Wuhan 430074, China

**Keywords:** graphene oxide, Pickering emulsion, interface activity, applications

## Abstract

Pickering emulsions, employing solid or colloidal particles rather than surfactants to stabilize the oil-water interface, have attracted considerable attention owing to their enhanced stability and the potential for designing functional materials. In particular, Graphene Oxide (GO) has emerged as an effective stabilizer for such emulsions, owing to its unique physicochemical properties. This review systematically outlines the stabilization mechanisms of GO-based Pickering emulsions, providing fundamental insights that support further development in the field. We comprehensively examine recent advances in the preparation and characterization of GO-stabilized emulsions and highlight their broad applications, including the synthesis of advanced materials and uses across various industrial sectors. Finally, we discuss current challenges and suggest promising directions for future research on GO-stabilized Pickering emulsions.

## 1. Introduction

Emulsions, crucial in various fields such as food, chemical, and materials preparation, are dispersion systems that homogenously mix two or more immiscible liquids. Traditional emulsions rely on surfactants or amphiphilic polymers to stabilize, thereby reducing the interfacial tension of the solution. However, this approach is plagued by problems like poor emulsion stability, excessive surfactants, and environmental unfriendliness [1]. The emulsions discovered a century ago by Ramsden [2] and Pickering [3], where solid particles irreversibly adsorbed at liquid-liquid or gas-liquid interfaces, demonstrated outstanding stability. Named Pickering emulsion, this emulsion has garnered significant attention from researchers. Compared to traditional emulsions, Pickering emulsions are characterized by their simplicity of preparation, high stability, biocompatibility, and responsiveness to external stimuli such as pH and temperature [4].

The selection of appropriate solid particles is essential for constructing high-performance Pickering emulsions. Factors such as particle size, shape, wettability, and surface chemistry collectively determine their behavior at the interface and the final emulsion properties [5]. Various inorganic materials like Laponite [6] and organic materials such as cellulose nanocrystals [7] and proteins [8] have been effectively used as Pickering stabilizers. In recent years, graphene oxide (GO), a two-dimensional (2D) nanomaterial comprising single atomic layers resulting from graphite oxidation, has offered exceptional advantages [9]. GO exhibits an amphiphilic structure, rendering it an optimal Pickering stabilizer [10]. Its basal plane consists of hydrophobic sp^2^-hybridized carbon atoms, while oxygen-containing functional groups (such as carboxyl, hydroxyl, and epoxy groups) on the edges and surfaces render it hydrophilic. These unique chemical properties allow GO to spontaneously adsorb and stabilize at oil-water interfaces, similar to surfactant molecules. Furthermore, the two-dimensional flexible sheet structure, large specific surface area, and ease of functionalization of GO offer extensive design possibilities in constructing structurally controllable and functionally diverse Pickering emulsion systems [4].

In recent years, significant progress has been made in research on Pickering emulsions stabilized by GO, with applications expanding from basic emulsion stabilization to advanced areas such as advanced materials preparation, energy storage, drug delivery, and environmental protection. For instance, Pickering emulsions stabilized by GO can serve as templates for the preparation of porous materials [11], for the synthesis of core-shell microcapsules via interfacial polymerization [12], or as microreactors for catalytic reactions [13]. Therefore, comprehending how the structural characteristics of GO determine its performance in Pickering emulsions is crucial for advancing this field. Despite the abundance of reviews on Pickering emulsions, there is a lack of a dedicated review specifically focusing on GO-based Pickering emulsions. This review aims to thoroughly analyze and evaluate the structural characteristics of GO, including their chemical structure, physical morphology, and surface chemistry, as well as their effectiveness in stabilizing Pickering emulsions. The review focuses on the GO-based Pickering emulsion’s interfacial behavior, stability control, and influencing factors, summarizes its use in materials preparation and various industrial fields over the past two decades, and prospects its potential in enhancing functional material design.

## 2. Basics of Pickering Emulsions

In recent decades, Pickering emulsions have gained significant attention due to their improved stability, multifunctional properties, and environmental friendliness compared to traditional surfactant-based systems [4]. This increased adoption has been driven by advancements in material science and nanoparticle engineering. Various solid particulates, such as emerging nanomaterials (e.g., metal-organic frameworks, covalent organic frameworks), inorganic compounds (e.g., silica, clays, GO, carbon dot, Laponite), and organic particles (e.g., protein, cellulose nanofibers, polysaccharide complex particles), have been deliberately designed as emulsion stabilizers [7,8,14,15,16,17,18,19,20,21]. As a result, Pickering emulsions now serve as the foundation for innovations in fields including cosmetics, pharmaceutical delivery, food science, enhanced oil recovery, and wastewater treatment.

### 2.1. Particles as Emulsifiers

Many properties of particles, such as amphiphilicity, particle concentration, size, and shape, significantly influence the characteristics of Pickering emulsions.

The amphiphilicity of particles may impact the anchoring strength of particles at the interface, as well as the stability and type of particle-stabilized emulsions. The amphiphilicity of particles is characterized by the three-phase contact angle *θ* [22]. The contact angle *θ* is determined by the Young’s equation:

(1) $$cos\theta =(\gamma_{SO}-\gamma_{SW})/\gamma_{OW}$$

where *γ*_so_ represents the solid/oil interfacial tension, *γ*_sw_ represents the solid/water interfacial tension, and *γ*_ow_ represents the oil/water interfacial tension.

**Figure 1:**
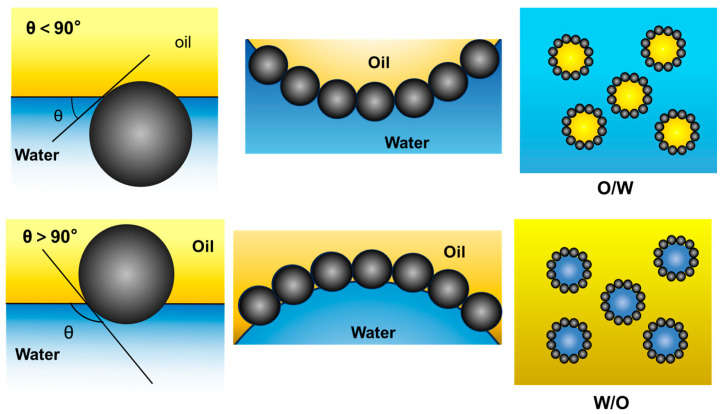
Position of a spherical particle at a planar oil-water interface with a specific contact angle, and corresponding probable positioning of particles at a curved oil-water interface.

Hydrophilic particles with a contact angle *θ* < 90° predominantly reside in the water phase, while hydrophobic particles with a contact angle *θ* > 90° predominantly reside in the oil phase [23], as illustrated in Figure 1. Consequently, hydrophilic particles exhibit a preference for the interfacial curvature of O/W emulsions, whereas hydrophobic particles exhibit a preference for the interfacial curvature of W/O emulsions. However, when particles are either too hydrophilic (low *θ*) or too hydrophobic (high *θ*), they tend to stay dispersed in either the aqueous or oil phase, respectively, leading to extremely unstable emulsions [24]. In addition, Janus particles, with one hydrophobic and one hydrophilic side, enhance emulsion stability by positioning their hydrophilic side in water and their hydrophobic side in oil [25]. Consequently, these amphiphilic Janus particles function as efficient emulsion stabilizers.

The particle concentration directly impacts the interfacial coverage and the final droplet size of emulsions. Typically, increasing particle concentration can provide more interfacial stabilizing material, thereby forming smaller and more stable droplets [6]. However, intriguingly, researchers have shown that even at low particle coverage, macroscopically stable emulsions can be achieved, possibly due to the Brownian motion of particles at the interface [26].

Particle size and morphology significantly impact the stability of emulsions. Generally, nanoparticles, with their higher surface area to volume ratio compared to microparticles, can adsorb more effectively at interfaces. The emulsion showed smaller droplet size and higher apparent viscosity as the nanoparticle size decreased [27]. The morphology of particles determines their packing at interfaces and the structure and mechanical properties of the interfacial film, thus influencing the ultimate stability of emulsions. Spherical particles are the earliest and most extensively studied particles in Pickering emulsions. Researchers have expanded the diversity of particles, such as dimeric, ellipsoidal, flaky, and rod-shaped particles, to produce more stable, multifunctional Pickering emulsions [22]. For instance, 2D flaky materials, including GO and montmorillonite nanosheets, are capable of forming dense covering layers at interfaces, which contribute to superior stability [28].

In addition to the individual characteristics of particles, interactions between particles at interfaces, e.g., electrostatic interactions, hydrogen bonding, hydrophobic interactions, significantly influence the macroscopic properties of emulsions. These interactions can lead to the formation of a network structure of particles at the interface, and even trigger gelation of emulsions, thereby greatly enhancing the mechanical strength and resistance to destabilization of the emulsion. For instance, Zou et al. [29] utilized hydrogen bonding interactions between zein and tannic acid to prepare complex colloidal particles. These particles not only stabilize the emulsion but also form a crosslinked network between droplets, ultimately forming a stable Pickering emulsion gel. Furthermore, using mixtures of two or more particles with different functional groups, opposite charges, or opposite wettabilities as stabilizers can enhance the stability of emulsions and impart them with new functionalities through synergistic effects [30].

### 2.2. Stabilization Mechanism of Pickering Emulsion

**Figure 2:**
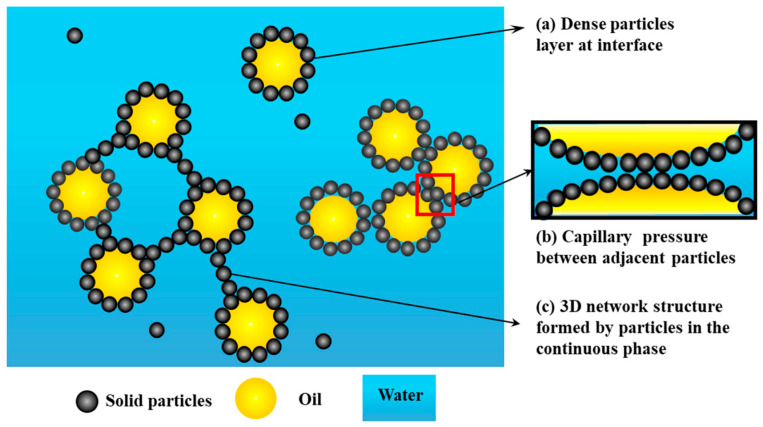
Stabilization mechanisms of Pickering emulsions. (**a**) Dense interfacial layer, (**b**) Capillary pressure between adjacent particles, and (**c**) 3D network structure in the continuous phase.

The stability of Pickering emulsions is primarily attributed to the formation of a robust physical barrier created by solid particles at the oil-water interface. Upon contact with the interface, the solid particles partially replace the oil-water interface with solid-oil and solid-water interfaces. The adsorption of particles onto the interface leads to a decrease in the system’s free energy, with the energy decrease (Δ*E*) representing the particle’s desorption energy. For spherical particles, the desorption energy can be expressed as:

(2) $$\Delta E=\pi R^{2}\gamma_{OW}(1\pm cos\theta {)}^{2}$$

where *R* is the particle radius, *γ*_ow_ is the interfacial tension between oil and water, and *θ* is the three-phase contact angle of the particle at the oil-water interface [4]. For micrometer or nanometer-sized particles when *θ* = 90°, even with low interfacial tension, their desorption energy significantly exceeds the particles’ thermal kinetic energy. Therefore, particle adsorption at the interface is considered irreversible [31]. The irreversibly adsorbed particles densely pack on the surface of the liquid droplets, forming a protective layer with high mechanical strength (Figure 2). This layer effectively prevents contact and coalescence between the droplets through the spatial hindrance effect [5]. Therefore, the stability of Pickering emulsions is often improved with a higher concentration of particles.

The high capillary pressure between adjacent particles at the interface is another factor that promotes the stability of Pickering emulsions [32]. Non-spherical particles, such as rod-like, disk-like, and fibers, can create more stable emulsion systems due to the increased capillary pressure between adjacent particles at the interface, which is enhanced by their anisotropic shapes [33].

Another important stabilization principle is the intrinsic ability of particles to act as versatile thickening agents and colloid stabilizer in the continuous phase. Agglomeration of particles like microgels, proteins, and rod-shaped particles occurs in the continuous phase, promoting the development of a network structure. This 3D network structure in the continuous phase plays a crucial role in stabilizing Pickering emulsions [34]. Hu et al. [35] prepared stable Pickering emulsions stabilized by gliadin colloidal particles. The microstructures, including the interfacial framework, particle distribution, and droplet state, were analyzed through optical and confocal laser scanning microscopy. The findings confirmed that, apart from Pickering stabilization, the network formed by the particles and the network based on dispersed droplets also played a role in stabilizing the emulsions.

## 3. Structure and Properties of GO

The excellent performance of GO as a stabilizer for Pickering emulsions is attributed to its amphiphilicity and unique hierarchical structure. These characteristics, from chemical composition to physical morphology, collectively determine the behavior of GO at the oil-water interface. Additionally, the excellent chemical reactivity of the functional groups on the GO surface endows it with a richer chemical behavior, imparting superior interfacial properties that are not present in traditional particle emulsifiers such as silica, clay, and polymeric particles.

### 3.1. 2D Sheet Structure

Following its successful isolation, graphene, characterized by its exceptional electrical, thermal, mechanical, and optical properties as a result of its 2D honeycomb lattice structure formed by closely stacked single-layer carbon atoms with sp^2^ hybridized orbitals, swiftly became a focal point of research in the fields of materials science and condensed matter physics [36]. However, the strong π-π attraction typically causes 2D graphene sheets to form stacked structures, limiting their processing and applications in solution. To address this limitation, researchers have developed various strategies, among which the preparation of GO through chemical oxidation is one of the most common and effective methods.

**Figure 3:**
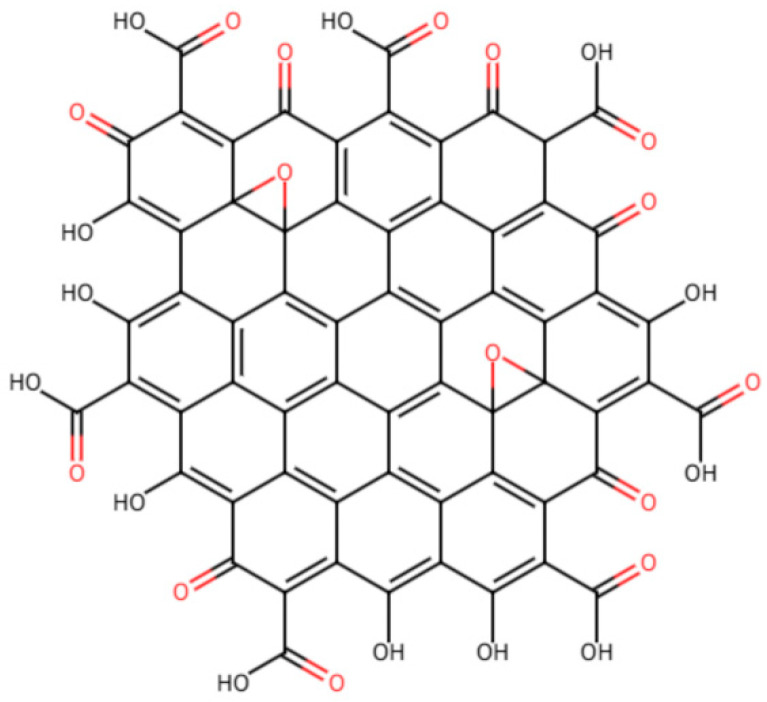
Schematic of the structure of a GO sheet.

The preparation of GO typically involves the Brodie, Staudenmaier, and Hummers methods, which entail treating graphite powder with strong oxidants (e.g., potassium permanganate) to introduce a significant amount of oxygen-containing functional groups onto the basal plane and edges of graphene [36]. The types and distribution of oxygen-containing functional groups determine the macroscopic properties of GO. According to widely accepted structural models (e.g., the Lerf–Klinowski model), the basal plane of GO is primarily populated by epoxy and hydroxyl groups (Figure 3), while the edges of the layers are enriched with carboxyl and carbonyl groups [37]. This structure results in the coexistence of sp^2^ hybridized graphene regions and sp^3^ hybridized oxygenated regions on the GO layers, creating a heterogeneous electronic and chemical structure.

### 3.2. Amphiphilicity and Interface Activity

Traditionally, due to its excellent dispersibility in water, GO has been widely believed to be completely hydrophilic. However, Kim et al. utilized Brewster angle microscopy to verify the amphiphilic properties and surface activity of GO sheets, demonstrating the presence of regionally distributed hydrophilic/hydrophobic characteristics in GO [9]. The amphiphilicity of GO arises from the chemical heterogeneity present in its 2D planar structure. The GO sheets exhibit strong hydrophilicity at their edges due to the high concentration of ionizable carboxyl groups, while the partially oxidized sp^2^ carbon regions on the basal plane maintain the hydrophobic properties of graphene. This distinctive structure confers GO sheets with characteristics of polymers, colloids, and amphiphilic molecules [38].

The amphiphilic nature of GO enables its spontaneous migration and adsorption to interfaces in water-oil biphasic systems, aiming to minimize the overall system’s free energy. Hence, GO demonstrates remarkable activity at the interface. The self-assembly behavior of GO at interfaces serves as the foundation for its emulsification properties and the construction of various ordered structures, such as, films, hydrogels, and hollow microspheres [10].

### 3.3. Functional Groups in GO

The incorporation of functional groups in GO leads to substantial modifications in its properties. Besides the enhanced hydrophilicity, GO exhibits decreased strength, conductivity, and thermal stability compared to graphene [39]. The Young’s modulus of monolayer GO decreases as determined by atomic force microscopy cantilever experiments [40]. This reduction is attributed to the presence of sp^3^ hybridized carbons and structural defects that weaken the strength of the carbon atomic framework. Although the individual strength of monolayer GO is inferior to that of graphene, macroscopic materials assembled from it, such as GO paper or films, exhibit excellent mechanical properties. This is mainly attributed to the robust hydrogen bonding network formed between the layers by oxygen functional groups and interlayer water molecules [41]. Due to the severe disruption of the sp^2^ conjugated network, GO is generally regarded as an electrical insulator. During heating, the oxygen-containing functional groups on the surface of GO decompose, typically showing significant mass loss starting around 200 °C. This characteristic serves as both a limitation, restricting its application in high-temperature environments, and an advantage, as heat treatment is an effective method for reducing GO and modulating its performance [42].

The functional groups on the GO surface offer abundant reaction sites for chemical modification and functionalization, making it a versatile platform [10]. For example, the presence of hydroxyl groups in GO enables the formation of hydrogen bonds with other hydroxyl-rich molecules. Additionally, the carboxyl groups located at the edges induce negative charges through the ionization of -COOH. Delocalized electrons spanning sp2-hybridized carbon atom domains introduce π-π interactions with other π-conjugated materials. The chemical composition and molecular structure of GO dictate its polymer-like properties.

### 3.4. Functionalized GO and Reduced GO

Despite being an excellent emulsifier, the emulsifying performance of pristine GO can still be further enhanced and customized. Chemical modification, known as functionalization, of the oxygen-containing functional groups on the surface of GO allows precise control of its surface properties, enabling the design of emulsifiers with specific functions and improved performance. Functionalized graphene oxide (fGO) obtained through further chemical modification by attaching specific functional groups or molecules onto the GO surface possesses enhanced functionality [43]. Covalent functionalization entails attaching functional groups directly to the oxygen-containing sites on the GO surface using chemical reactions like amidation, esterification, or diazonium chemistry. The resulting covalently fGO demonstrates enhanced stability and improved compatibility with various matrices, facilitating its seamless integration into composite materials or specific applications [39]. In addition, non-covalent functionalization entails the adsorption or intercalation of molecules onto the GO surface through weak interactions, including hydrogen bonding, electrostatic interactions, or π-π stacking [43]. This approach facilitates reversible functionalization and maintains the intrinsic properties of GO. The properties of non-covalently fGO can be readily modified by varying the adsorbed molecules, allowing for customization of its attributes for specific applications.

Another crucial characteristic of GO is its regulability. The removal of oxygen functional groups, either partially or entirely, through chemical, thermal, or photochemical means, leads to the formation of reduced GO (rGO). This process facilitates the gradual restoration of graphene’s sp^2^ conjugated structure, thereby allowing for precise modulation of its electrical, optical, and thermal properties [44]. Therefore, GO is not only a unique material but also a material system that can be tailored according to specific application requirements.

## 4. Factors Affecting the Performance of Pickering Emulsions Stabilized by GO

At the liquid-liquid interface, amphiphilic GO can spontaneously adsorb, driven by the minimization of free energy, thus facilitating self-assembly and rendering it suitable for stabilizing Pickering emulsions [45]. The GO sheets adsorbed at the interface form a robust mechanical barrier, physically isolating adjacent droplets and effectively preventing their coalescence and Ostwald ripening. Furthermore, GO sheets adsorbed at the interface can crosslink or overlap, forming a two-dimensional network structure that further enhances the mechanical strength and elasticity of the interfacial membrane, enabling the droplets to resist deformation and rupture [12]. Therefore, Pickering emulsions stabilized by GO typically exhibit excellent long-term stability, remaining stable for several months or even longer without phase separation [9].

GO demonstrates excellent performance in stabilizing Pickering emulsions due to its unique structural characteristics. The performance of emulsions is influenced by the intrinsic properties of GO, external environmental conditions, and emulsion system parameters. A thorough understanding of these factors is essential for precise control of the emulsifying properties based on GO.

### 4.1. Inherent Characteristics of GO

GO is not a substance with a completely fixed chemical composition and structure; rather, its properties largely depend on the preparation method and post-treatment processes, which directly influence its behavior at oil-water interfaces.

The degree of oxidation in GO, characterized by the type and quantity of oxygen-containing functional groups, is crucial in determining its contact angle. Various preparation methods result in GO with different C/O ratios and functional group distributions. A comparative study revealed that GO prepared via the Staudenmaier method contains a higher concentration of carbonyl groups, enhancing its polarity, thus improving its hydrophilicity. Conversely, GO produced by the Hummers method exhibits larger sheet sizes [46]. These property differences directly impact the wetting behavior of GO, its adsorption at interfaces, and the type and stability of the resulting emulsions.

The type of Pickering emulsion stabilized by GO is primarily determined by the wetting properties of the stabilizing particles. Due to its high content of oxygen-containing functional groups, pristine GO typically exhibits good hydrophilicity, thereby tending to stabilize O/W type emulsions [47]. However, the wettability of GO can be regulated through chemical modifications or reduction. For instance, hydrophobic GO can be achieved by grafting hydrophobic chains such as alkyl or fluoroalkyl chains to stabilize W/O emulsions [48]. Luo et al. [49] successfully prepared various types of emulsions, including Ionic Liquid (IL)-in-oil and oil-in-IL, by alkylating or perfluoroalkylating GO, demonstrating the feasibility of controlling emulsion types by modulating the surface chemistry of GO.

GO tends to localize at the oil-water interface, showing a slight preference towards the aqueous phase, thereby forming a steric barrier that segregates the oil and water phases. Alberto et al. [50] evaluated the interfacial activity of functionalized GO through Molecular Dynamics simulations. The chemical functionalization of GO may induce a synergistic effect on its behavior, leading to the acquisition of properties characteristic of a molecular surfactant. This is evidenced by a shift in the orientation of GO sheets from parallel to perpendicular, resembling the behavior of typical “head-tail” molecular surfactants [50]. Therefore, the interfacial activity of GO can be regulated by modulating its surface functional groups, thereby altering its dispersibility and promoting emulsion formation [50].

### 4.2. Chemical Environment

The physico-chemical parameters of the emulsion, such as pH and ionic strength, significantly influence the interfacial behavior of GO and the overall stability of the emulsion.

The pH primarily regulates the performance of GO by affecting the protonation/deprotonation state of carboxyl groups (-COOH) on its surface [51]. At low pH, carboxyl groups mainly exist in the -COOH form, resulting in weaker electrostatic repulsion between GO sheets, facilitating aggregation but potentially enhancing hydrogen bonding for a tighter interfacial network. Under acidic conditions, stable GO emulsions typically exhibit improved stability [52]. At high pH, carboxyl groups deprotonate to -COO^−^, rendering GO sheets negatively charged, thereby enhancing electrostatic repulsion for better dispersion in the aqueous phase. However, excessive electrostatic repulsion may hinder close stacking of layers at the interface. When the pH approaches the isoelectric point of GO, the surface charge decreases, leading to particle aggregation, which may result in coarsening or destabilization of emulsion droplets [53]. Therefore, pH could serve as an external switch effectively regulating the stability of GO emulsions.

The presence of electrolytes in aqueous solution affects the double layer surrounding GO sheets. The addition of salt ions shields the negative charges on the GO sheet surface, compresses the double layer, and weakens the electrostatic repulsion between the sheets. This leads to the aggregation of GO sheets in the aqueous phase or at interfaces [52]. At low salt concentrations, this effect may contribute to the formation of denser interfacial films, enhancing emulsion stability. However, at high salt concentrations, strong shielding effects can cause extensive aggregation of GO, disrupting emulsion stability and potentially leading to phase separation or transformation.

### 4.3. Emulsion System

In addition to the GO characteristics and the external chemical environment, the formulation and preparation process of the emulsion, such as the concentration of GO, the type of oil phase, and preparation methods, is also crucial.

The concentration of GO is a key factor influencing the size and stability of emulsion droplets [54]. Typically, within a certain range, an increase in GO concentration leads to more GO sheets available to stabilize the interface [55]. This allows for faster coverage of newly formed oil-water interfaces, resulting in smaller and more uniform droplets. Research has shown that the average droplet size decreases and the stability of the emulsion improves with increasing GO concentration [52]. However, excessively high GO concentrations may significantly increase the viscosity of the water phase or cause aggregation of GO sheets in the water phase, which could hinder the formation and stability of the emulsion.

The polarity and interaction forces between the oil phase and GO play crucial roles in the formation and stability of emulsions [56]. Aromatic oils, such as toluene and styrene, containing aromatic rings, demonstrate a heightened affinity towards GO’s sp2 carbon framework via π-π interactions, which can impact the orientation and adsorption strength of GO at the interface [52]. Non-polar oil emulsions stabilized by GO were successfully formed, whereas emulsion formation was impeded by polar oil phases [57].

Emulsion preparation typically requires energy input to disperse droplets and promote the migration of GO to the interface. Common techniques include ultrasonication and high-speed shearing. The intensity and duration of energy input, such as ultrasonication time, directly impact the initial droplet size distribution. Prolonged ultrasonication or increased energy input generally leads to smaller droplets, but excessive energy input can disrupt the GO interfacial film [52]. Hence, optimizing process parameters is essential for achieving the desired emulsion quality.

## 5. Advanced Materials Prepared via GO-Based Pickering Emulsion

**Figure 4:**
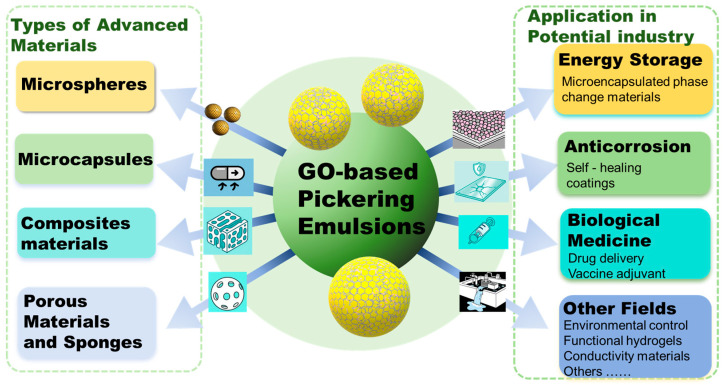
Types and applications of materials prepared via GO-based Pickering emulsion.

Stable Pickering emulsions stabilized by GO serve not only as stable dispersion systems but also as versatile microreactors and templates. It is possible to accurately synthesize structurally controlled advanced materials, including polymer composites, microcapsules, and porous scaffolds (Figure 4). The emulsion droplets serve as microsphere molds, while the interfacial GO sheets act as both structural templates and functional components. The remarkable stability of GO-based Pickering emulsions, akin to other particle-stabilized emulsions, is attributed to the irreversible adsorption of GO at the oil-water interface. This characteristic ensures the preservation of emulsion templates in the fabrication of advanced materials, thereby maintaining the integrity of droplet structures for precise replication of material porosity [21].

### 5.1. Microspheres

The polymer microspheres with excellent dispersion can be effectively achieved through Pickering emulsion polymerization. By using GO as the stabilizer, the polymerization of a monomer (the oil phase) results in polymer particles that are intimately coated with GO sheets, preventing the agglomeration that often plagues melt-mixing or solution-casting methods [45,58]. Stable polystyrene (PS) colloidal microspheres have been successfully synthesized by polymerizing styrene in a GO-stabilized Pickering emulsion [47]. Similarly, a polymer-functionalized GO microsphere prepared through Pickering emulsion polymerization [59] has been reported. The preparation methods of these microspheres are simple, and they are expected to be applied in the electromechanical, selective removal of heavy metal ions, and coating industries. Furthermore, microspheres with special properties and structures can be produced through the utilization of multiple Pickering emulsions [60]. For instance, a double Pickering emulsion stabilized by modified GO was designed to fabricate hollow imprinted microspheres for the detection of L-Cysteine [61]. Typically synthesized through polymerization, these microspheres find extensive applications in adsorption, catalysis support, and drug delivery, owing to their large interfacial area, low density, and robust mechanical characteristics [60].

### 5.2. Microcapsules

Templates for microcapsule fabrication have been created using Pickering emulsion stabilized by GO. GO adsorbed at the oil-water interface can undergo interfacial polymerization or cross-linking to create a robust shell encapsulating the internal functional substances. These functional microcapsules have been explored for applications in environmental protection, self-healing coatings, thermal insulation, and other fields.

Utilizing Pickering emulsions stabilized by GO as templates, Luo et al. [62] achieved the encapsulation of an ionic liquid through interfacial polymerization of GO. The resulting microcapsules exhibit efficacy in the removal of phenol pollutants from oil substances. Yu et al. [63] designed a one-component photo-responsive self-healing microcapsule (BAEA@GOMCs) based on Bisphenol A epoxy acrylate resin emulsion stabilized by GO. BAEA@GOMCs were embedded in a waterborne epoxy matrix to form a self-healing composite coating on hot-dip galvanized steel surfaces. Similarly, Yan et al. [12] employed the Pickering emulsion template method combined with interfacial polymerization to fabricate GO/polyurethane/polyaniline organic-inorganic hybrid shell microcapsules utilizing isophorone diisocyanate as the core material. The method for preparing hybrid shell microcapsules is efficient, simple, and environmentally friendly. The microcapsules exhibit excellent dispersion, small size, and a strong hybrid shell. Multifunctional microcapsules with paraffin as core material and PS/modified GO as shell material were prepared via Pickering emulsion polymerization [64]. The microcapsules were blended with waterborne silicone resin to form a coating that enhanced the waterproofing, anti-static electricity, and thermal insulation properties of the fabric.

Various other specialized functional microcapsules are also widely investigated. Yan et al. [65] reported a facile method to encapsulate ammonium dinitramide (ADN) using Pickering emulsion as a soft template, and alkylated GO as a particle surfactant. The results showed that GO significantly suppressed the high hygroscopicity and exhibited remarkable catalytic efficiency in the thermal decomposition of ADN. These outstanding properties make the microcapsule a promising alternative for use in the fabrication of high energetic, low signature and eco-friendly propellants. Wang et al. [66] prepared capsule shells with hindered urea bonds through interfacial polymerization within an oil-in-oil Pickering emulsion stabilized by fGO. Upon isolation and gentle heating, the shells exhibit the ability to either merge into monoliths or disintegrate. These dynamic shells offer a pathway to manipulate the morphology of composite materials, with potential applications in energy storage, separations, additive manufacturing, controlled release, and others.

### 5.3. Composites Materials

High-performance composite materials containing GO can be fabricated using a GO-based Pickering emulsion template strategy. GO, in Pickering emulsion, not only serves as the stabilizer but also endows materials with exceptional properties. It has been demonstrated that the thermal stability and mechanical rigidity of poly(methyl methacrylate) (PMMA)/GO composites, prepared via Pickering emulsion polymerization, are significantly improved even at a very low GO content (0.3 wt%) [45]. Likewise, researchers have successfully synthesized GO-stabilized PS colloidal particles [47] and GO-PS nanocomposites with specific optical properties [67]. Recently, Fe_3_O_4_@GO-PS composite particles prepared using this approach have shown potential in the field of electro/magneto-rheology [68]. Interestingly, Bian et al. [69] introduced a novel method for synthesizing hollow composite materials through in situ interfacial growth of nanoparticles in Pickering emulsion. By inducing the growth of ZIF-8 nanoparticles at the oil-water interface in the Pickering emulsion stabilized by GO, hollow ZIF-8/GO composites were successfully fabricated.

By applying Pickering emulsions onto the surfaces, composite materials can acquire distinct functional properties. Epoxy resin emulsions stabilized by GO have been employed as a sizing agent for carbon fibers [70]. This emulsion facilitates the deposition of a uniform film of epoxy and GO onto the fiber surface, which significantly enhanced the interfacial adhesion between the carbon fibers and the bulk epoxy matrix, leading to composites with superior interlaminar shear strength. These methods offer a novel approach to preparing various types of specific composite, nanoparticle composite materials, and hollow composite materials.

### 5.4. Porous Materials and Sponges

Upon removal of the continuous or dispersed phase from stable high internal phase emulsions stabilized by GO, porous materials or aerogels with interconnected pore structures can be obtained. These materials have garnered significant attention owing to their high specific surface area, low density, and exceptional adsorption properties.

Jahandideh et al. [11] reported a polymer-free emulsion templating method where GO and cellulose nanocrystals (CNC) were used as stabilizers to prepare a robust porous sponge by removing the oil phase after GO reduction, demonstrating its excellent performance in pollutant adsorption. Zhang et al. [71] prepared lightweight, elastic, and conductive graphene porous aerogels with a multiscale structure through a versatile Pickering emulsion-based approach. The unique multiscale structure enhances electron transport and load transfer, resulting in exceptional mechanical and electromagnetic interference (EMI) shielding capabilities. Remarkably, the gel-like rheological properties of the emulsion enable the fabrication of ultralight graphene scaffolds with customizable geometries through 3D printing. This study presents a versatile approach for producing ultralight and highly elastic graphene aerogels with outstanding EMI shielding performance, demonstrating wide-ranging potential applications across various industries.

## 6. Applications in Potential Industry

Functional materials prepared via GO-based Pickering emulsion template method have shown promising applications in various fields. Compared to other particle emulsifiers, GO stands out for its remarkable adsorption and barrier properties, abundant surface-active groups that enhance its chemical reactivity, and the excellent electrical and thermal conductivity of rGO [36,37]. Thus, GO serves not only as a superior particle emulsifier but also as an intelligent, multifunctional emulsifier, uniquely combining top-tier stabilization capability with unprecedented functional integration. GO-based emulsions are pivotal precursors for functional materials. In this section, we focus on discussing the applications of these materials in energy storage, anticorrosion, biological medicine, environmental pollution control, and other emerging areas.

### 6.1. Energy Storage

Microencapsulated phase change material (MEPCM) is an efficient thermal energy storage material. Graphene-based materials have ultrahigh thermal conductivity and have been used as thermal conductive enhancement materials in MEPCMs [72]. The in situ polymerization method is the most widely adopted for the preparation of graphene-based microcapsules, and GO-enhanced MEPCM could generate the best morphology and smooth surface.

Wei et al. [73] prepared multifunctional phase change microcapsules with paraffin as the core and GO and lead tungstate as the double-shell through Pickering emulsion stabilized by GO. The microcapsules demonstrated high phase change reliability, thermal stability, anti-seepage performance, superhydrophobic properties, and good gamma-ray shielding properties. Similarly, Maithya et al. [74] prepared MEPCM with eicosane as the phase change material, polyurea as the shell, and GO as the stabilizer through Pickering suspension polymerization. Shao et al. [75] reported an organic-inorganic composite phase change material via Pickering emulsion polymerization. This material was a shape-stable hybrid emulsion gel that seamlessly combined sodium acetate trihydrate in the water phase with paraffin wax in the oil phase. Not only does GO act as an emulsifier, but its addition also improved the optical absorption properties of the material, leading to increased photothermal conversion efficiency. This method paves a broad avenue for combining organic and inorganic PCMs, which is an ideal choice for effectively utilizing solar energy and building energy storage.

### 6.2. Anticorrosion

Self-healing coatings based on microcapsules have garnered significant attention from researchers [76]. Microcapsules represent the predominant carrier material for fabricating advanced smart coatings due to their straightforward preparation process, exceptional sealing and mechanical properties, and customizability. A crucial requirement for microcapsules is a robust shell with excellent barrier properties and stability. Recently, the utilization of GO as a stabilizer in the emulsion polymerization for the synthesis of polymer/GO composite microcapsules has been a focal point of research in the anticorrosion field.

Wu et al. [28] synthesized two Pickering emulsions containing polystyrene acrylate-polysiloxane core-shell structures stabilized by GO. The GO interface enhanced the molecular configuration and crosslinking degree of the core-shell structure, promoting the formation of robust shielding films and a stable hydrophobic layer on cement hydrates. The Pickering coating films applied to cement exhibit high corrosion potentials against chloride and sulfate attacks, demonstrating resistance to aggressive ions and free radicals during acid/alkaline corrosion and photo-oxidative aging. This effectively prevents degradation of polymer molecules and adhesion failure on cementitious materials. Similarly, waterborne polyacrylic anticorrosion coatings were synthesized through Pickering emulsion stabilized by sulfonated reduced GO, exhibiting excellent dispersibility, compatibility and anticorrosive properties [28].

Zhang et al. [76] prepared hybrid microcapsules loaded with self-healing agent (linseed oil) synthesized by the Pickering emulsion template method. GO was used as a stabilizer, and the emulsion droplets contained aniline and linseed oil. After the polymerization of aniline, a unique GO/polyaniline hybrid shell was created, integrating the superior barrier capabilities of GO with the anti-corrosive properties of polyaniline. Electrochemical impedance spectroscopy and salt spray tests both confirmed that integrating microcapsules markedly enhanced the anticorrosion properties of water-borne coatings. Similarly, self-healing microcapsules, with a GO/polyurethane/polyaniline organic-inorganic hybrid shell, were synthesized through Pickering emulsion stabilized by GO, utilizing isophorone diisocyanate as the core material. Upon mechanical damage, the microcapsules ruptured to release the healing agent, which was triggered to cure and fill cracks under light exposure, enabling self-repair of the coating [12].

Numerous anticorrosive coatings [28,77,78,79,80,81] have also been investigated and utilized on various material surfaces, such as metals, concrete, wood, and plastics. These coatings are essential in industries like petrochemicals, construction, marine and offshore engineering, automotive and transportation, and municipal infrastructure. By creating protective barriers against corrosive agents, these coatings effectively prolong the lifespan of substrates in diverse environments.

### 6.3. Biological Medicine

Stable Pickering emulsions, known for their potential biocompatibility and unique carrier functions, have garnered increasing attention in biomedical applications such as drug delivery and tissue engineering.

Pickering emulsions stabilized by GO can serve as templates to encapsulate drugs in the core, creating drug carriers with GO shells. The abundant functional groups on the GO shell offer potential for subsequent targeted molecular modifications. Wang et al. [82] prepared a pH responsible polyacrylamide hydrogel loaded with doxorubicin hydrochloride via Pickering emulsion template, where the Pickering emulsion was stabilized by GO and modified hydroxyethyl cellulose. The release behavior of the hydrogel varies under different pH values, and the released doxorubicin hydrochloride remains biologically active in killing cancer cells. Zhao et al. [55] developed a GO-stabilized Pickering emulsion to serve as an adjuvant for boosting the immune response to the Chlamydia trachomatis Pgp3 recombinant vaccine. This emulsion, devoid of traditional surfactants, significantly enhances both humoral and cellular immunity, leading to mitigation of chlamydial-induced tissue damage in the genital tract.

Osteoarthritis is a common chronic degenerative condition that impacts the joints. Wu et al. [83] prepared an angelica essential oil Pickering emulsion stabilized by GO, and the emulsion was used as a joint injection lubricant. The prepared Pickering emulsion exhibited superior lubrication properties, with a 19% greater reduction in friction at the natural cartilage interface compared to hyaluronic acid, which is commonly used in osteoarthritis treatment. Furthermore, the Pickering emulsion demonstrated antioxidant activity and cell biocompatibility, indicating promising potential for clinical application.

### 6.4. Other Fields

The materials prepared using the GO-Pickering emulsion template demonstrate significant potential for applications in environmental pollution control, owing to their unique structural advantages and design flexibility. Edgehouse et al. [84] developed a method to encapsulate poly(α-olefins) within a polymer-based shell using a Pickering emulsion stabilized by GO and interfacial polymerization. The prepared capsules exhibit the ability to effectively eliminate various low molecular weight organic contaminants, including benzene, toluene, ethylbenzene, and p-xylene, from water. When a contaminated aqueous solution is passed through a column filled with these capsules, the contaminants are removed, and clean water is collected as the eluent. Jahandideh et al. [11] presented a new approach for producing emulsion-templated hybrid sponges suitable for efficient contaminant removal. GO and cellulose nanocrystals were utilized to stabilize the Pickering emulsions. Following this, GO was reduced with vitamin C, and the oil phase was extracted through multiple washing and boiling steps to create the sponge. The sponge was effective for removing contaminants in diverse water chemistries and exceeded the performance of granular activated carbon.

The electrical insulating properties of GO limit its applications in conductive composite materials, energy storage, and electrocatalysis. To restore graphene’s excellent electrical, thermal, and mechanical properties, the in situ reduction of GO to rGO at the interface addresses the issue of rGO’s strong hydrophobicity, enabling its enhanced interfacial adsorption [85]. This advancement can further broaden the applications of GO-based emulsions in various fields. Reducing agents such as hydrazine hydrate, sodium borohydride, and ascorbic acid, as well as thermal reduction methods, have been employed to reduce GO in GO-based emulsions or composite materials [11,86]. Isari et al. [85] presented a method for fabricating multifunctional rGO aerogels through structured emulsion templating, enabling customizable multiscale porosity. These aerogels exhibit exceptional performance in electromagnetic interference shielding, surpassing the specific shielding effectiveness of previously reported mate-rials significantly. Tran et al. [87] prepared polymer/GO nanocomposite latexes via emulsion polymerization. The latex films were subsequently annealed to convert GO to rGO, thereby imparting excellent conductivity to the composite films.

Furthermore, materials prepared using the stable GO Pickering emulsion templating method also find wide applications. For instance, Qi et al. [68] synthesized Fe_3_O_4_@GO-PS composite particles with responsiveness to electric and magnetic fields via Pickering emulsion polymerization. Hu et al. [88] prepared a green and multifunctional Janus nanosheet, which was prepared by decorating each side of GO with hydrophilic tannic acid-titanium complex and hydrophobic PS chains. Lak et al. [89] prepared a non-aqueous Pickering emulsions stabilized by alkylated GO, followed by the deposition of solutions of commodity polymers onto the droplet sur-faces, forming robust shells. This method offers a unique advantage in accessing capsules with a pure core composition, allowing for versatility in core and shell materials and enabling customization of capsule composition for specific applications. Otherwise, a self-assembled hybrid of amphipathic hydroxyethyl cellulose and GO was synthesized and employed as Pickering emulsifiers for the fabrication of functional porous hydrogels through the removal of the oil phase post-emulsion templating polymerization [90].

## 7. Conclusions and Outlook

Pickering emulsions have garnered significant interest in recent decades due to their simple preparation, exceptional stability, and reduced environmental impact compared to traditional emulsions stabilized by surfactants [91,92]. The unique interfacial architecture formed by particle monolayers provides robust resistance against coalescence and Ostwald ripening, making these systems ideal for applications ranging from food science and pharmaceuticals to advanced materials synthesis [93,94,95]. GO, as a prominent 2D nanomaterial, has significantly advanced the field of emulsions due to its unique properties [9,10]. With its unique amphiphilicity, large specific surface area, and remarkable mechanical strength, GO serves as an excellent stabilizer for Pickering emulsions. Its ability to form a robust and flexible protective layer at the oil-water interface enhances the stability of emulsions. Moreover, the characteristics of GO can be modulated by external factors like pH and ionic strength, paving the way for the development of responsive emulsions. Additionally, the outstanding physicochemical properties of GO, including conductivity, photothermal conversion capability, and mechanical robustness, can be transferred to emulsions, enabling the integration of multiple functions on a single material platform. The versatility and adjustability of GO offer numerous opportunities for applications in diverse fields such as material templating [96], energy storage [72], and catalysis [97], demonstrating its capability to address practical challenges.

Utilizing GO-based Pickering emulsion as a functional material preparation platform highlights its distinctive advantages and vast potential. This platform enables the production of diverse material forms, including microsphere, microcapsules, porous bulk materials, and composite materials. Precise control over the materials’ microstructure, such as pore size, porosity, and connectivity, can be achieved by adjusting the emulsion formulation and preparation conditions [91], facilitating the synthesis of multifunctional composite materials. Moreover, the mild process conditions employed in this approach align with the principles of green chemistry.

Despite the numerous achievements in advanced materials prepared via GO-based Pickering emulsions, significant challenges remain in the precise control of GO sheets, scalability of production, recyclability, and long-term biocompatibility under complex environmental conditions. The high cost of GO hinders its large-scale application, as its high-quality preparation still relies on chemical methods using strong oxidants. Maintaining consistency in product quality during large-scale production remains problematic, with variations in structure and properties between batches affecting high-end applications. Developing a green, low-cost, and consistently high-quality GO production technology is crucial for its practical utilization. Despite significant challenges, the outstanding performance and market demand for GO have propelled the development of related enterprises. For instance, The Sixth Element (Changzhou) Materials Technology has the capacity to produce 11 million tons of GO annually, and it is anticipated that more companies will be able to supply high-quality GO products in the future. In addition, while GO is generally considered safe, its long-term behavior in complex environments, degradation products, and potential ecological impacts require thorough and systematic evaluation. Particularly in biomedical applications, prioritizing the assessment of its biocompatibility and potential toxicity is essential. These challenges represent the obstacles faced by GO-based emulsions in material synthesis and various industries, necessitating collaborative efforts across different fields.

In summary, the use of GO-based Pickering emulsions as templating platforms represents a robust and versatile pathway for advanced functional materials. While challenges remain in scaling production, elucidating fundamental mechanisms, and ensuring biocompatibility, progress in interdisciplinary research and synergy with advanced manufacturing methods are paving the way for broader application. The development of intelligent, multifunctional, and precision-engineered material systems based on GO Pickering emulsions holds strong potential to address critical global issues in energy sustainability, environmental remediation, and healthcare.

## Data Availability

No new data were created or analyzed in this study. Data sharing is not applicable to this article.
